# Supplementary material for: Gtf2i-encoded transcription factor Tfii-i regulates myelination via Sox10 and Mbp regulatory elements
Source: Nat Commun. 2025 Sep 26;16:8518. doi: 10.1038/s41467-025-63500-4 (PMC12474925; doi:10.1038/s41467-025-63500-4)
Supplement: Supplementary file 1 — Supplementary Information [file 41467_2025_63500_MOESM1_ESM.pdf]

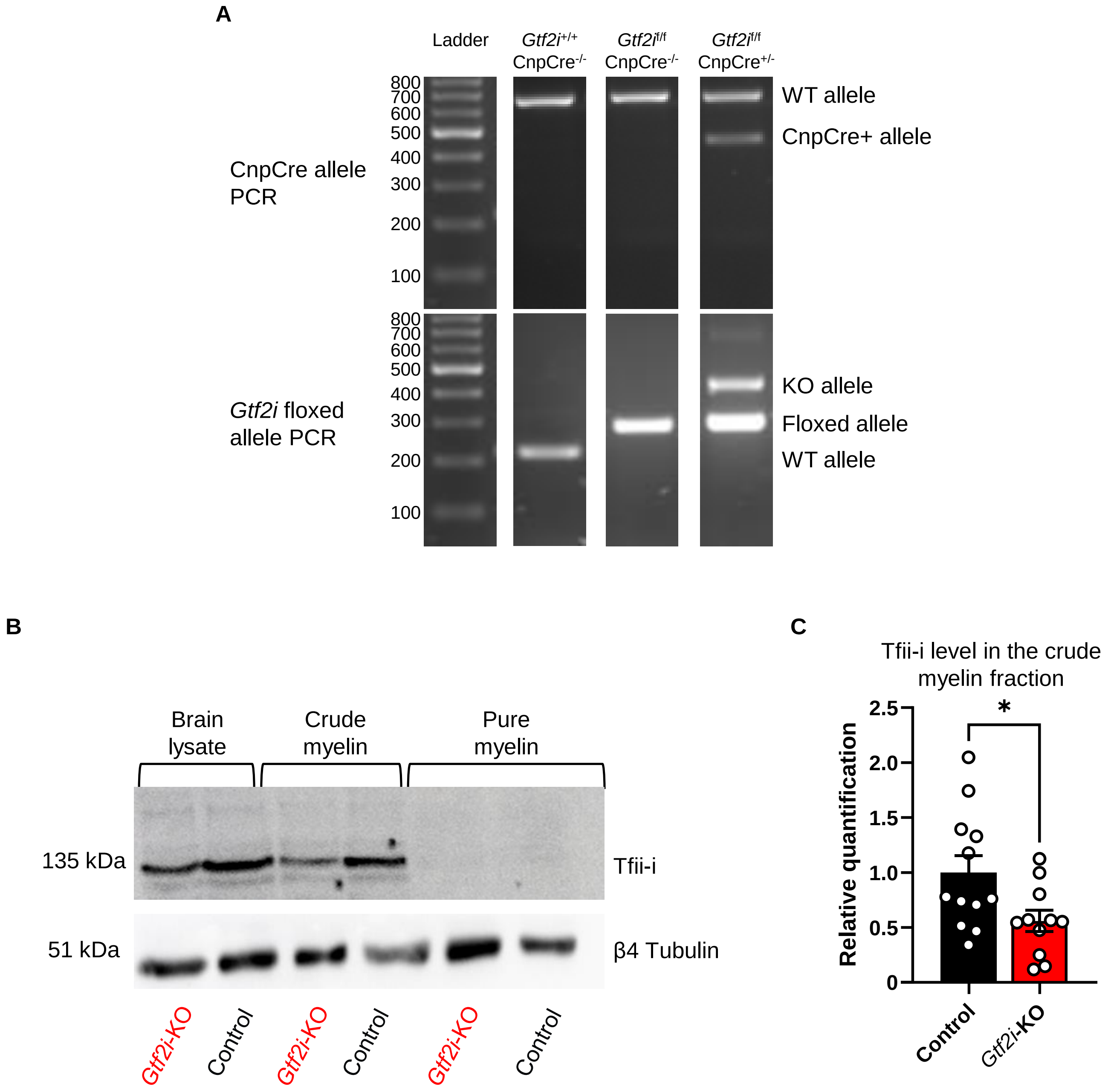

<u>**Supplementary figure 1. Generation of a mouse model with specific deletion of *Gtf2i* from myelinating glia.**</u> **(A)** Representative images of CnpCre^{+} allele and *Gtf2i* floxed allele PCR. Left lane - WT mouse (*Gtf2i*$^{+/+}$, CnpCre$^{-/-}$). Middle lane - littermate control (*Gtf2i*$^{f/f}$, CnpCre$^{-/-}$). Right lane - *Gtf2i*-KO mouse (*Gtf2i*$^{f/f}$, CnpCre$^{+/-}$). Upper bracket – CnpCre^{+} allele PCR. Upper band – WT allele, lower band – CnpCre^{+} allele. Lower bracket – *Gtf2i* floxed allele PCR. Upper band – KO allele, middle band – floxed allele, lower band – WT allele. *Gtf2i*-KO mice show PCR products of a KO allele from CnpCre^{+} cells and floxed allele from other cell types in the *Gtf2i* floxed allele PCR. **(B)** Western blot assay demonstrating Tfii-i expression levels in the brain lysate, crude and pure myelin fraction. No Tfii-i expression was detected in the pure myelin fraction derived from either control or *Gtf2i*-KO mice. **(C)** Significantly reduced Tfii-i expression in the crude myelin fraction of *Gtf2i*-KO mice compared to controls (n=12 control, n=11 *Gtf2i*-KO, two-sided t-test, P=0.0278). Data are presented as mean values ± SEM. Control levels normalized to 1. Protein levels were normalized to β-tubulin IV. * $P < 0.05$. Source data are provided as a Source Data file.

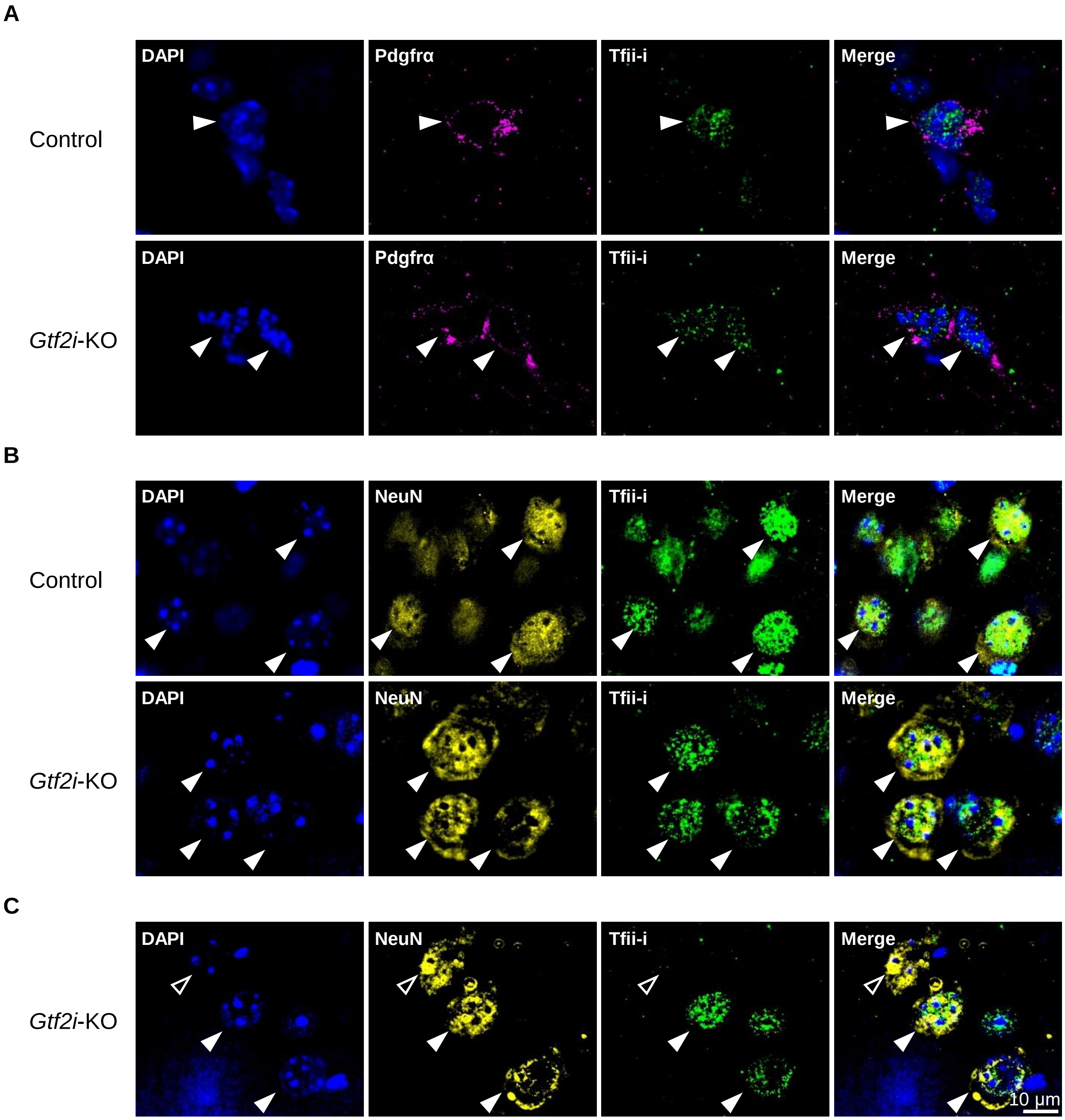

Supplementary figure 2. Verification of expression in different cell types of the CNS. **(A, B)** Representative image of immunofluorescence assay showing intact Tfii-i expression in **(A)** OPCs and **(B)** neurons, in the motor cortex of both control (upper bracket) and *Gtf2i*-KO mice (lower bracket). **(C)** Representative images of immunofluorescence assay shows the absence of Tfii-i in a NeuN$^+$ cell, at the motor cortex of a *Gtf2i*-KO mouse (black arrowhead). The absence of Tfii-i from NeuN$^+$ cells was remarkably rare and appeared in a very small number of cells.

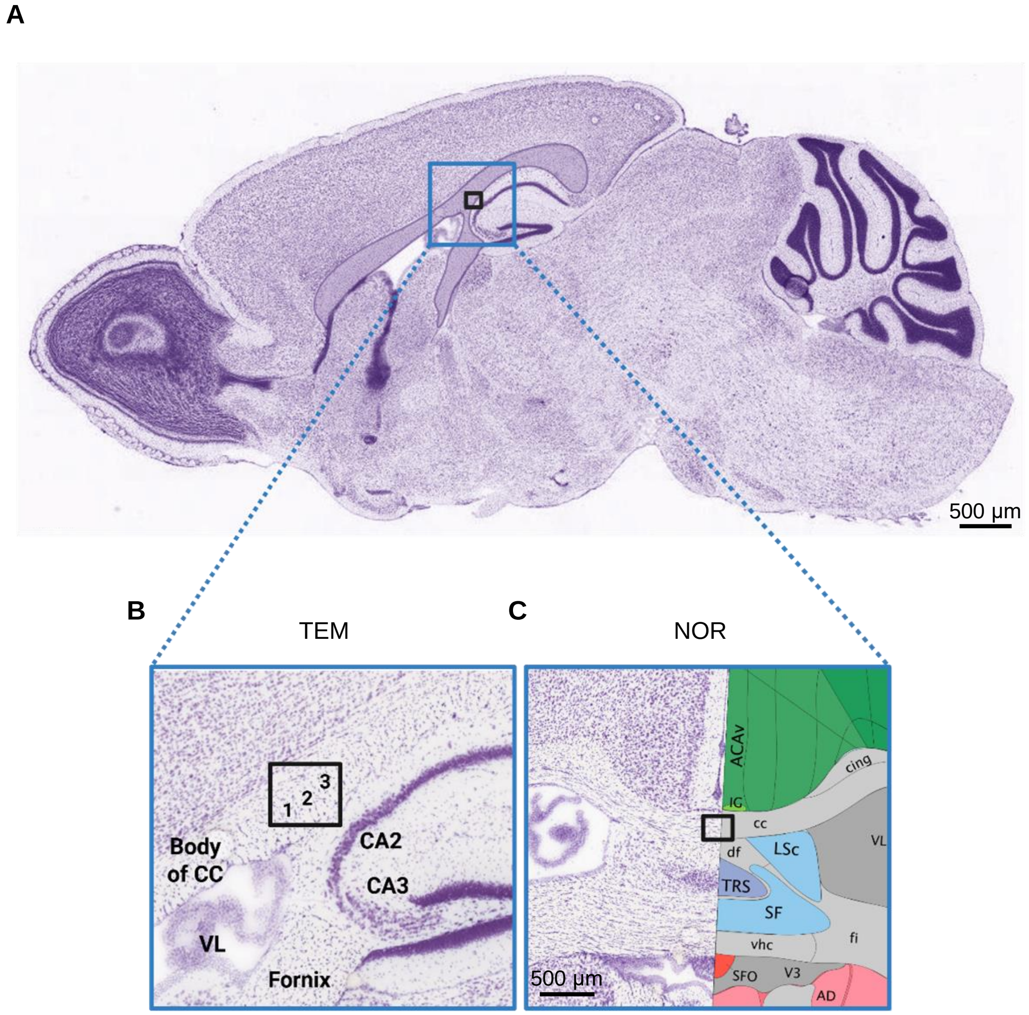

Supplementary figure 3. Anatomic location of TEM and NOR experiments in the midline of the CC. **(A)** Midsagittal section with the CC and fornix highlighted (light purple). TEM samples were taken from the CC at the level of the fornix, marked as a black square in the midsagittal section. **(B)** Zoom-in image of the anatomic location from which TEM samples were taken. The numbers 1-3 delineates our TEM image acquisition strategy where three different locations along the rostro-caudal and dorso-ventral axis of the CC were quantified, per sample. **(C)** Coronal section taken from the same bregma and depicts the location in which images for NOR properties quantification were taken.

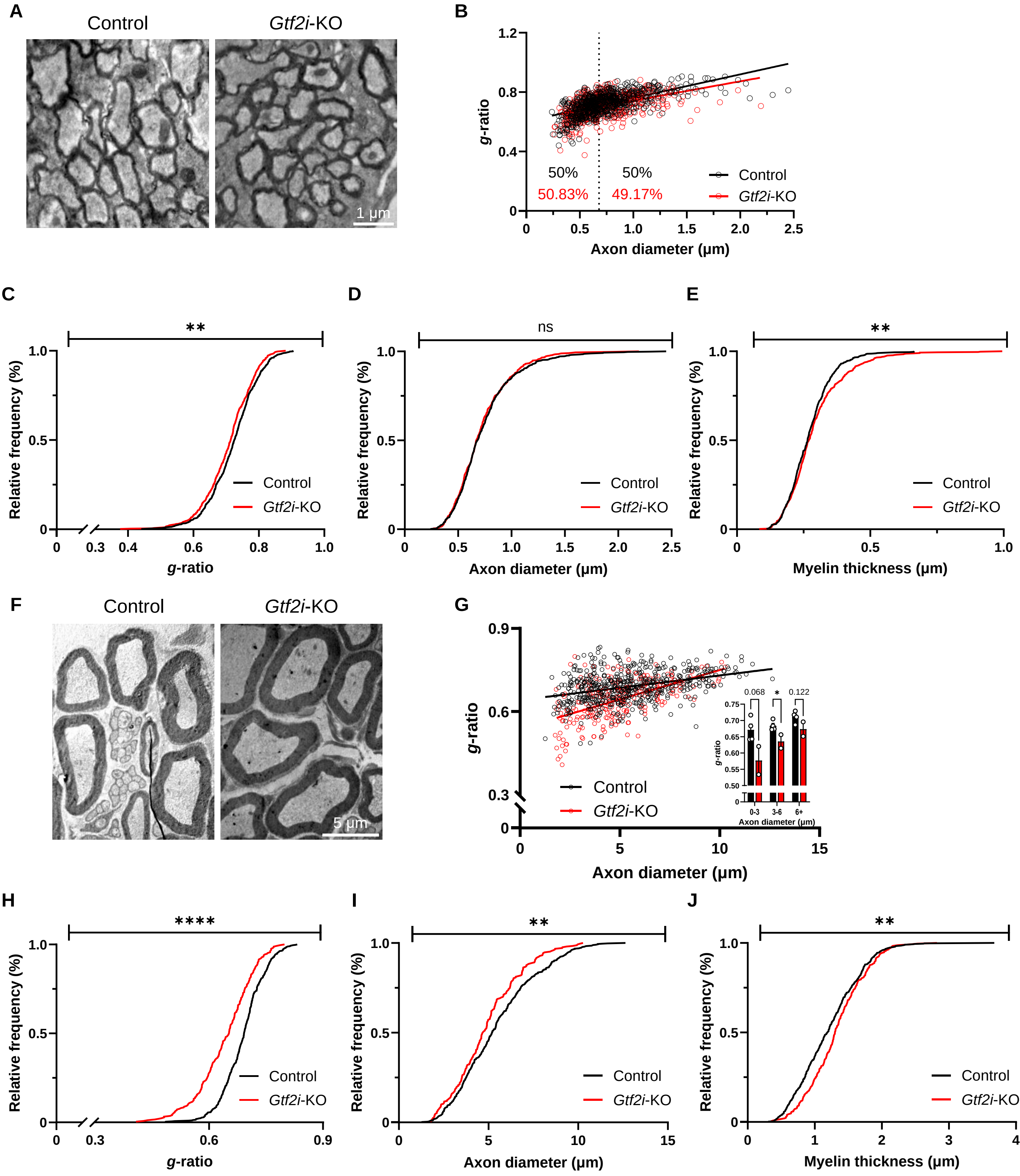

Supplementary figure 4. Hypermyelination of the CC and SN of *Gtf2i*-KO mice persists through adulthood. (A) Representative TEM images of axons from the CC of control and *Gtf2i*-KO P90 mice. **(B)** Scatter plot of *g*-ratio values and their respective axon diameters in the CC of P90 *Gtf2i*-KO and control mice. The dashed line indicates the median diameter of control mice axons (0.68µm), while the numbers on each side of the line represent the percentage of axons below (left) and above (right) this value for each genotype (two-sided simple linear regression, slopes *P*=0.0265). **(C)** *Gtf2i*-KO P90 mice axons in the CC present with significantly lower *g*-ratio values compared to controls (*P*=0.0075). **(D)** Myelinated axon diameter distribution is unchanged between P90 *Gtf2i*-KO and control mice in the CC (*P*=0.75). **(E)** Myelin thickness of P90 *Gtf2i*-KO mice axons in the CC is significantly increased, as compared to controls (*P*=0.0069). **(F)** Representative TEM images of axons from the SN of control and *Gtf2i*-KO P90 mice. **(G)** Scatter plot of *g*-ratio values and their respective axon diameters. *Gtf2i*-KO P90 mice SN axons present with lower *g*-ratio values, compared to controls (two-sided t-test, *P*=0.068, 0.048, 0.122, for axons with diameters of 0-3µm, 3-6µm, and 6+µm, respectively. Data are presented as mean values ± SEM.). **(H)** P90 *Gtf2i*-KO mice axons in the SN present with significantly lower *g*-ratio values, compared to controls (P=$1.146*10^{-12}$). **(I)** Smaller axonal diameters are observed in the SN of P90 *Gtf2i*-KO mice, compared to controls (*P*=0.0034). **(J)** Myelin thickness of P90 *Gtf2i*-KO mice axons in the SN is significantly increased, as compared to controls (*P*=0.0013). **(B-E)** *n*=4 control, 706 axons. *n*=3 *Gtf2i*-KO, 543 axons. **(G-I)** *n*=3 control, 540 axons. *n*=2 *Gtf2i*-KO, 250 axons. **(C-E, H-J)** Two-sided Kolmogorov-Smirnov test. ns – non-significant, ** $P < 0.01$, **** $P < 0.0001$. Source data are provided as a Source Data file.

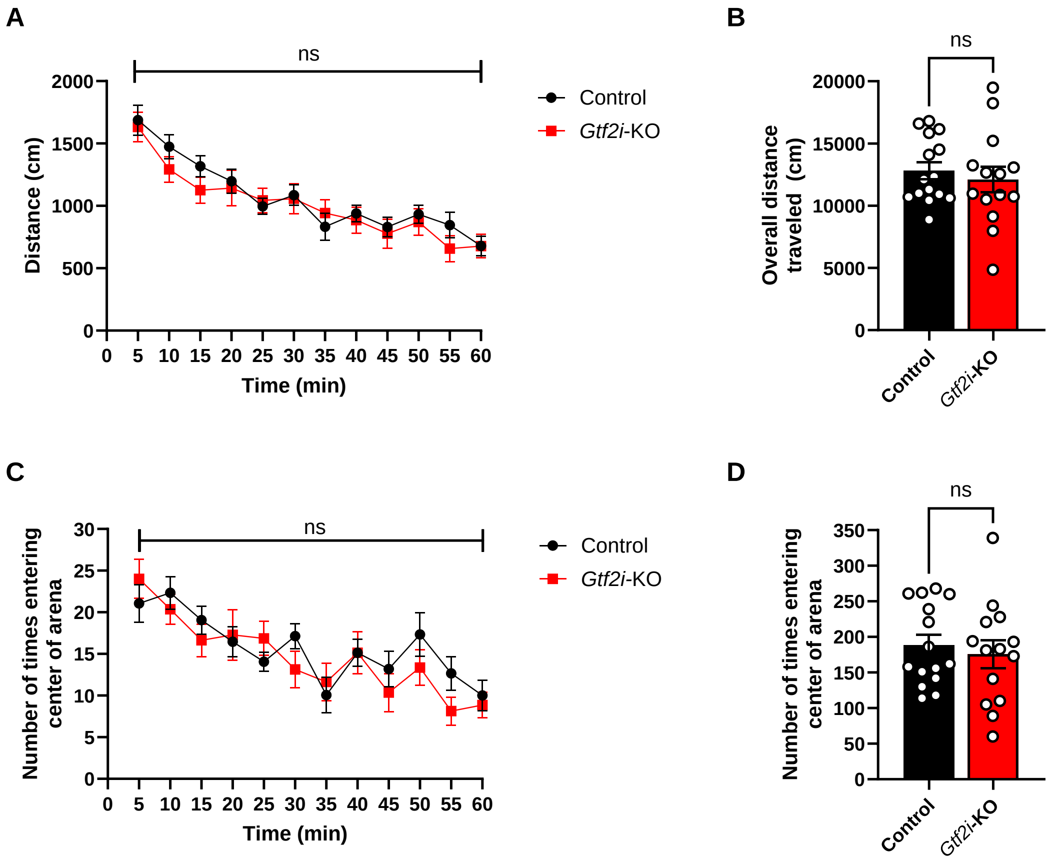

Supplementary figure 5. Distance traveled and the number of entries to the center of the arena are unchanged in the open-field test.
(A) Distance traveled by time bins (two-sided two-way ANOVA, *P*=0.56) and **(B)** overall distance traveled (two-sided t-test, *P*=0.56) during the open-field test are unchanged in *Gtf2i*-KO mice compared to controls. **(C)** The number of entries into the center of the arena in the open-field test, **(C)** divided by time bins (two-sided mixed-effects analysis *P*=0.58), and **(D)** overall number of entries (two-sided t-test, *P*=0.6) are unchanged in *Gtf2i*-KO mice compared to controls. **(A-D)** *n*=15 control, *n*=14 *Gtf2i*-KO. Data are presented as mean values ± SEM. ns – non-significant. Source data are provided as a Source Data file.

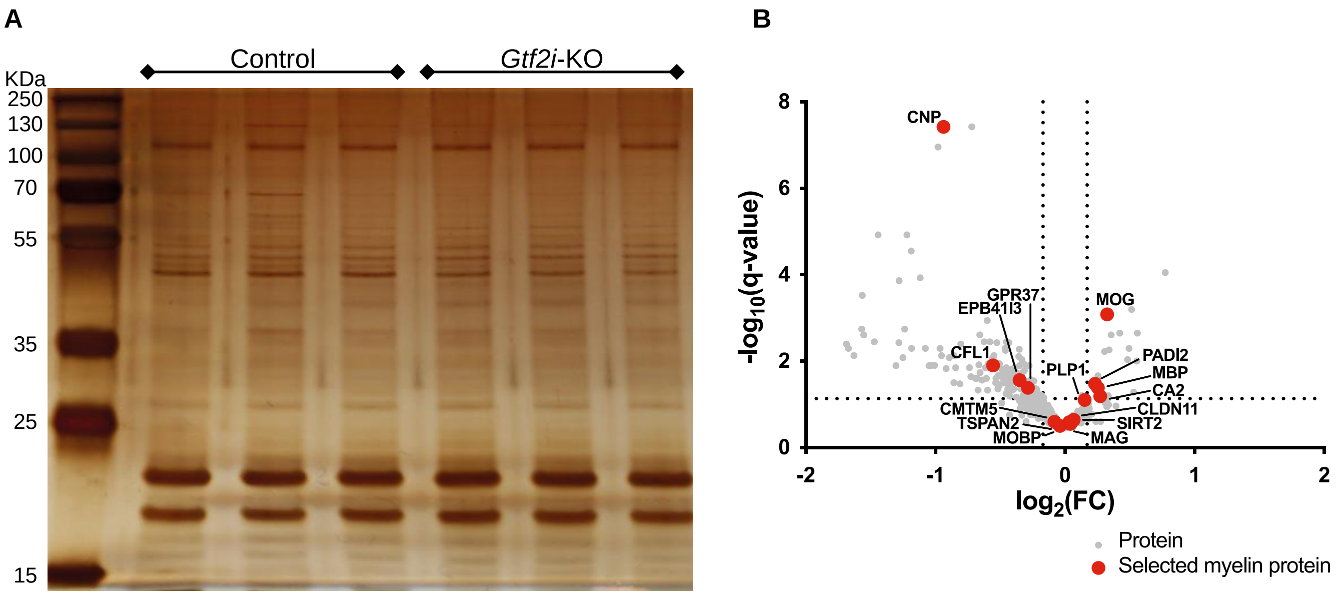

<u>Supplementary figure 6. Analysis of myelin purified from the brains of *Gtf2i*-KO and control mice</u>. (A) Silver gel analysis of myelin purified from the brains of *Gtf2i*-KO mice shows overall similar pattern and intensity of bands in the myelin fraction compared to controls. Shown are three biological replicates per genotype. **(B)** Quantitative proteome analysis of brain myelin comparing myelin composition in *Gtf2i*-KO and control mice. Analyzed were n=3 mice per genotype and four technical replicates per mouse. The Volcano plot displays data points representing log_2-fold change and $-log_{10}$-transformed q-values of 551 identified proteins in *Gtf2i*-KO compared to control myelin. Red dots highlight selected known myelin proteins. Stippled lines indicate thresholds. See Figure 4A for heatmap and Supplementary Data 1 for entire dataset. Source data are provided as a Source Data file.

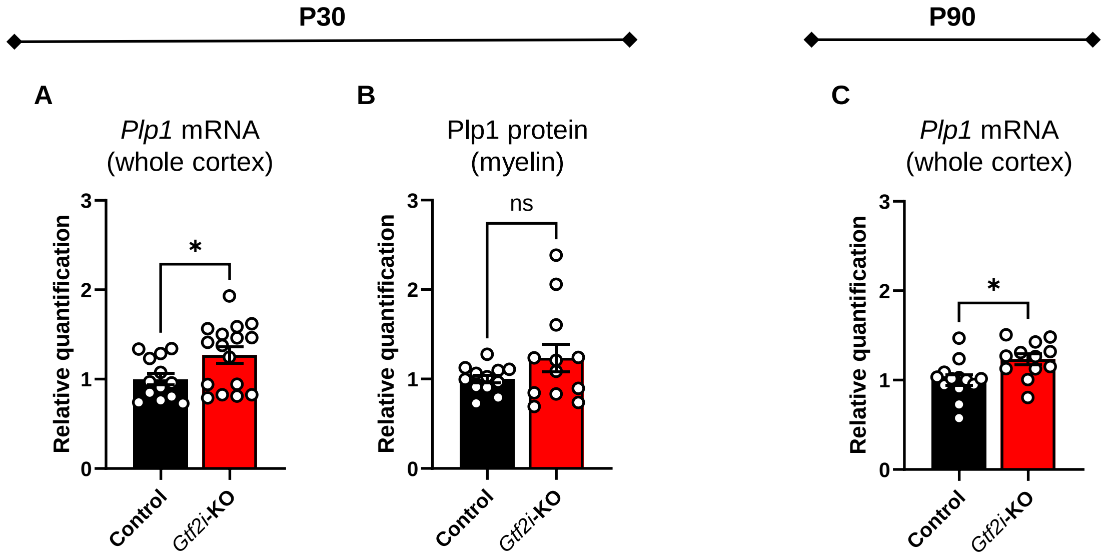

Supplementary figure 7. Additional molecular alterations in myelin-related genes and proteins in *Gtf2i*-KO mice. **(A)** Whole cortex *Plp1* mRNA level is significantly elevated in *Gtf2i*-KO P30 mice, compared to controls (*n*=13 control, *n*=16 *Gtf2i*-KO, two-sided t-test, *P*=0.028). **(B)** Plp1 protein level is unchanged in the myelin of *Gtf2i*-KO mice, compared to controls (*n*=12 control, *n*=12 *Gtf2i*-KO, two-sided Welch's t-test, *P*=0.165). **(C)** Whole cortex *Plp1* mRNA level is significantly elevated in *Gtf2i*-KO P90 mice, compared to controls (*n*=13 control, *n*=12 *Gtf2i*-KO, two-sided t-test, *P*=0.011). Data are presented as mean values ± SEM. ns – non-significant, * $P < 0.05$. Source data are provided as a Source Data file.

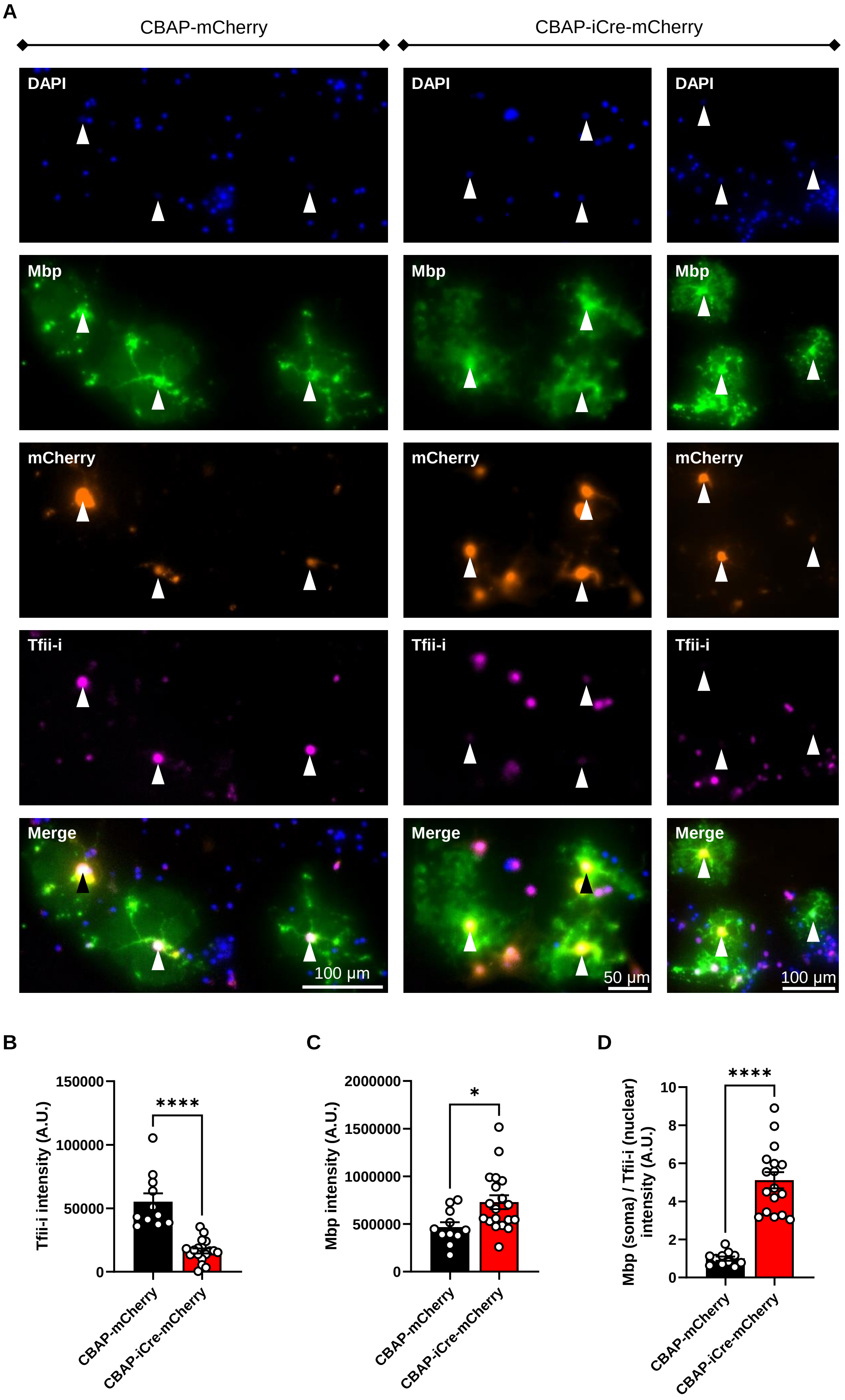

Supplementary figure 8. Exogenous deletion of *Gtf2i in-vitro* in an OL-enriched primary cell culture. (A) Representative images of an AAV-infected OL-enriched primary cell cultures. CBAP-mCherry or CBAP-iCre-mCherry AAVs were added to differentiated OL-enriched cell culture. While not all cells were transduced, the integrity of the culture and cells were unchanged following infection with either AAV or in the absence of transduction. **(B-C)** Intensity measurements from an OL-enriched cell culture following the introduction of control or iCre-expressing AAV. *n*=3 control (11 cells overall), *n*=3 iCre (19 cells overall). Measurements of Tfii-i and Mbp intensity were taken from the same cell. **(B)** Tfii-i intensity levels are significantly reduced following the introduction of an iCre-expressing AAV (two-sided t-test, $P=1.387*10^{-7}$) while **(C)** Mbp intensity levels are significantly increased (two-sided t-test, $P=0.0156$). **(D)** The ratio between Mbp intensity levels (soma) and Tfii-i intensity levels (nuclear) is significantly higher following the introduction of an iCre-expressing AAV (two-sided t-test, $P=3.75*10^{-8}$). Control levels normalized to 1. Data are presented as mean values ± SEM. * $P < 0.05$, **** $P < 0.0001$. Source data are provided as a Source Data file.

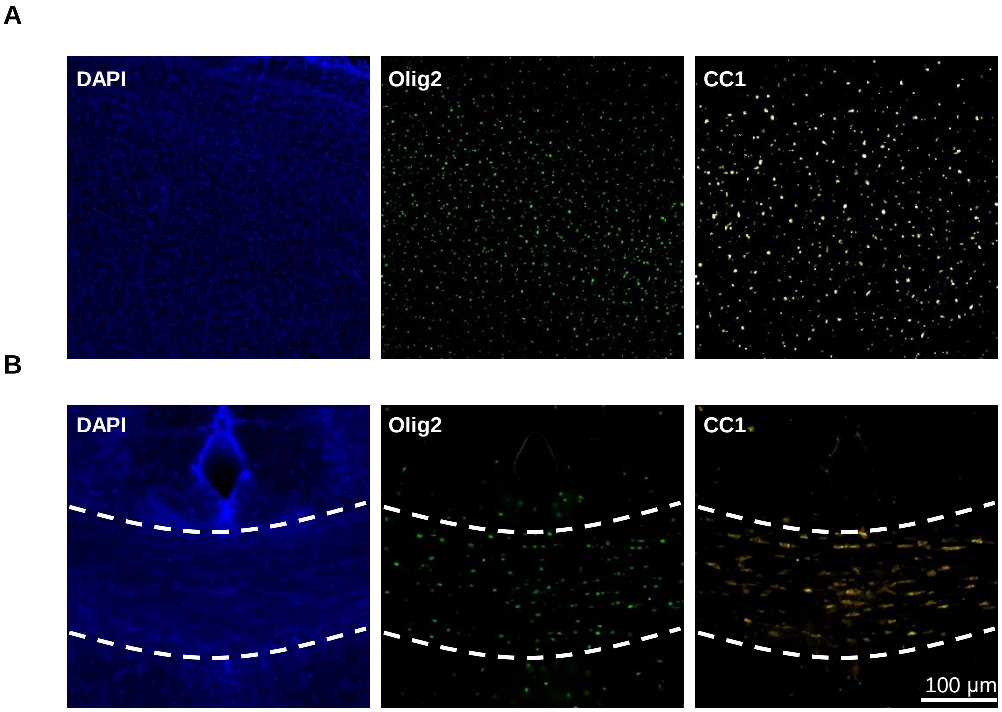

<u>Supplementary figure 9. Representative images of colocalization experiments.</u> **(A, B)** Representative image of immunofluorescence assay for colocalization experiments of mOLs from the **(A)** motor cortex and **(B)** CC. The dashed lines at **(B)** indicate the area in which cells were counted.

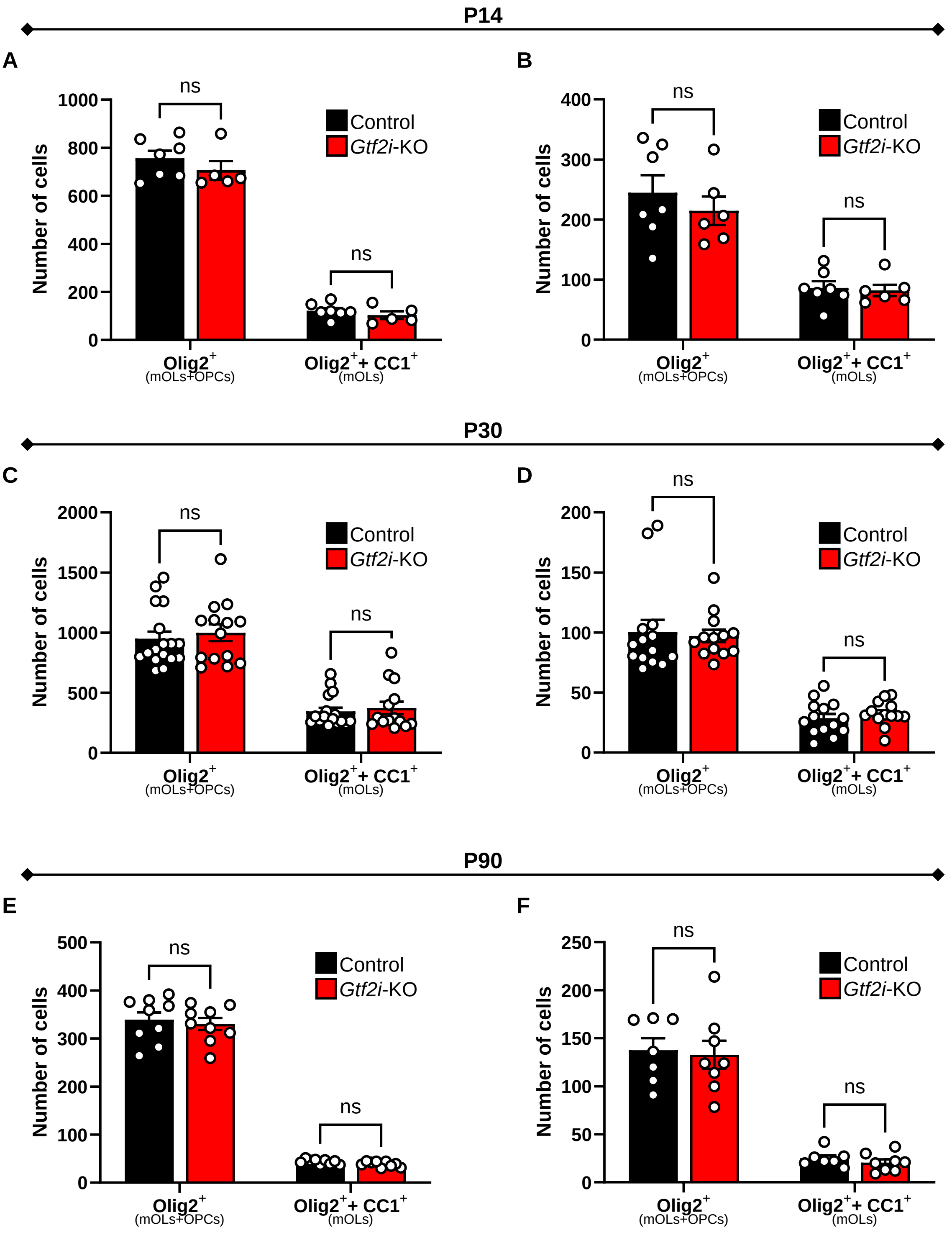

<u>Supplementary figure 10. Unchanged oligodendroglial cellular properties in *Gtf2i*-KO mice.</u> **(A-B)** Unchanged number of cells from the oligodendroglial lineage (Olig2⁺ cells) and mOLs (Olig2⁺ + CC1⁺ cells) in the **(A)** motor cortex (*n*=7 control, *n*=5 *Gtf2i*-KO. Olig2⁺ - *P*=0.33. mOLs - *P*=0.35) and **(B)** CC (*n*=7 control, *n*=6 *Gtf2i*-KO. Olig2⁺ - *P*=0.45. mOLs - *P*=0.78) of P14 *Gtf2i*-KO mice, compared to controls. **(C-D)** Unchanged number of Olig2⁺ cells and mOLs in the **(C)** motor cortex (*n*=17 control, *n*=14 *Gtf2i*-KO. Olig2⁺ - *P*=0.59, mOLs – *P*=0.62) and **(D)** CC (*n*=14 control, *n*=13 *Gtf2i*-KO. Olig2⁺ - *P*=0.79, mOLs – *P*=0.41) of P30 *Gtf2i*-KO mice, compared to controls. **(E-F)** Unchanged number of Olig2⁺ cells and mOLs in the **(E)** motor cortex (*n*=9 control, *n*=9 *Gtf2i*-KO. Olig2⁺ - *P*=0.65, mOLs – *P*=0.07) and **(F)** CC (*n*=7 control, *n*=8 *Gtf2i*-KO. Olig2⁺ - *P*=0.81, mOLs – *P*=0.37) of P90 *Gtf2i*-KO mice, compared to controls. **(A-F)** Two-sided t-test. Data are presented as mean values ± SEM. ns – non-significant. Source data are provided as a Source Data file.

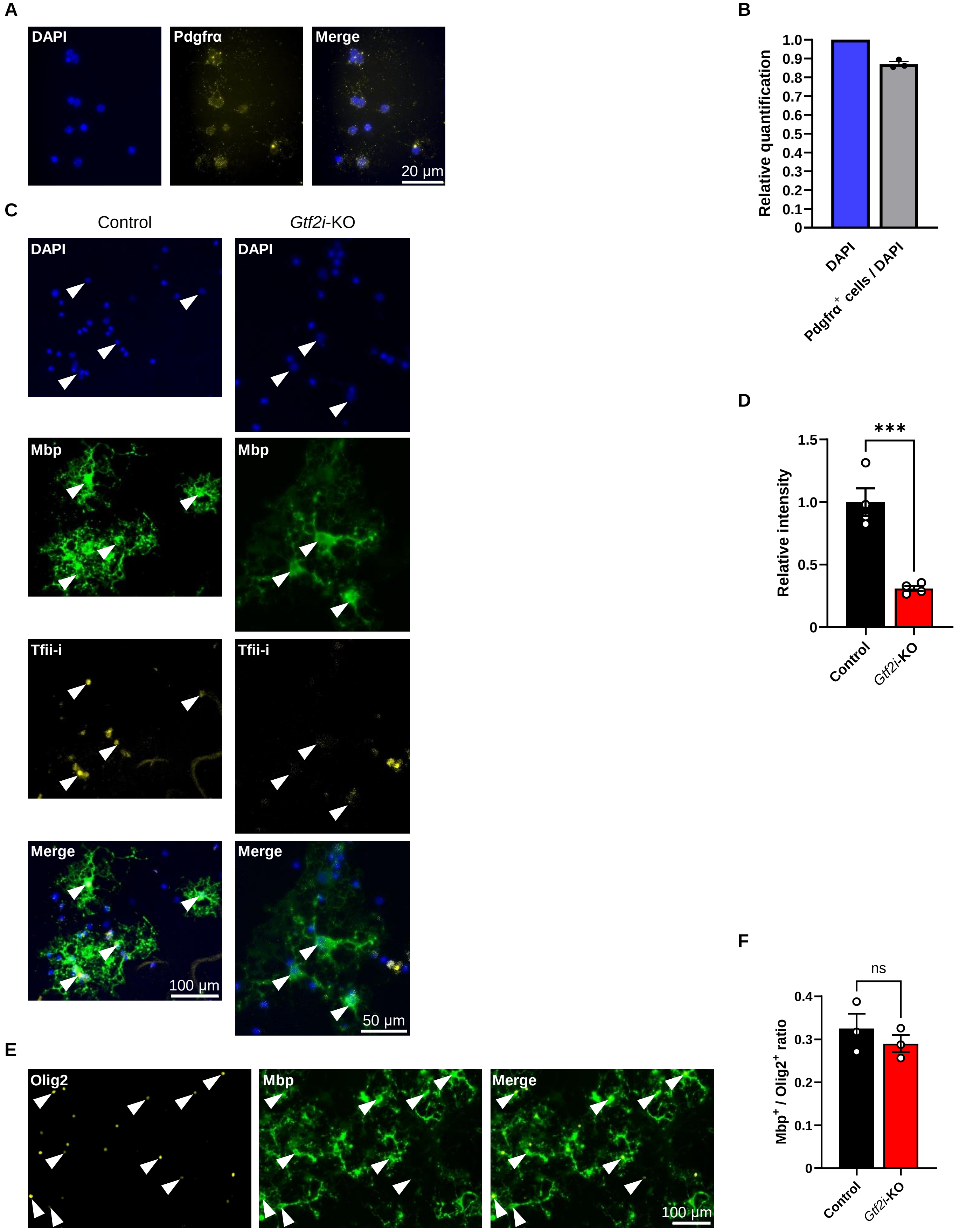

Supplementary figure 11. Primary cell cultures are OPC enriched and Tfii-i levels are decreased in Mbp⁺ cells derived from *Gtf2i*-KO mice while differentiated cell numbers are unchanged. **(A)** Representative image of immunofluorescence assay showing OPC enrichment in primary cultures derived from *Gtf2i*$^{f/f}$ mice. **(B)** Over 87% of DAPI⁺ cells are Pdgfrα⁺ in the OPC enriched culture at DIV3 (*n*=3 mice, 263-629 cells analyzed from each mouse). **(C)** Representative images of immunofluorescence assay showing Tfii-i expression in differentiated mOLs culture (Mbp⁺ cells), in control and *Gtf2i*-KO mice. **(D)** Tfii-i intensity is significantly decreased in Mbp⁺ cells of *Gtf2i*-KO mice, compared to controls (*n*=4, 45-56 cells analyzed from each mouse, two-sided t-test, *P*=0.0008). **(E)** Representative image of immunofluorescence assay for Olig2 and Mbp colocalization experiment, *in-vitro*. **(F)** Differentiation rate of OPCs into OLs on DIV4 following OPC isolation is unchanged between *Gtf2i*-KO and control mice, as indicated by similar Mbp⁺ / (Mbp⁺ + Olig2⁺) cell ratio (*n*=3, 584-1338 cells analyzed from each mouse, two-sided t-test, *P*=0.419). Data are presented as mean values ± SEM. ns – non-significant, *** $P < 0.001$. Source data are provided as a Source Data file.

A

Peak calling per replicate

Sample	Control 1	Control 3	Control 4	Control 5	Control 6
**# of peaks**	247621	198351	134244	250514	188152

B

Tfii-i peaks shared across replicates

Number of peaks	Shared across
26	5 samples
485	4 samples
9959	3 samples

C

Inter-replicate variability of Tfii-i binding in mOLs at the *Sox10* genomic locus

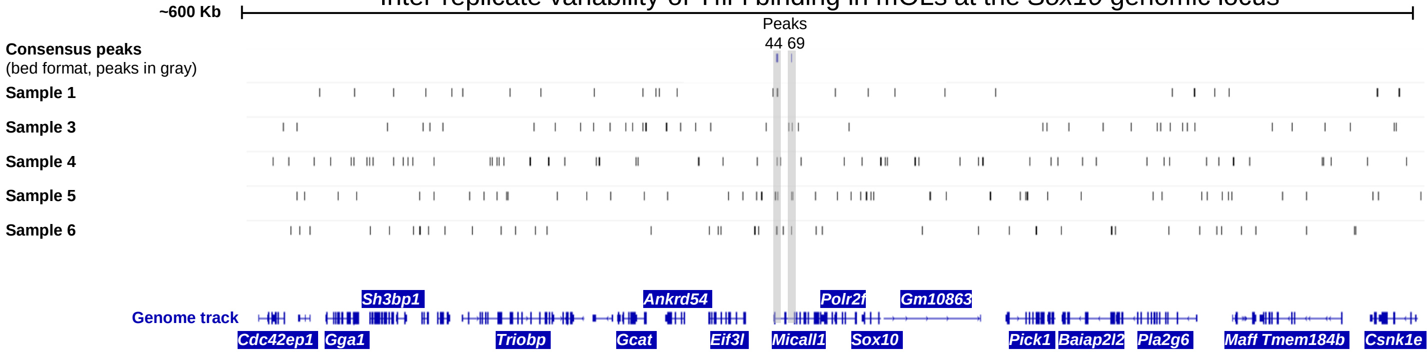

D

Overlap of Tfii-i, Olig2, and Sox10 binding in mOLs (*Tfb2m*/*Cnst* locus)

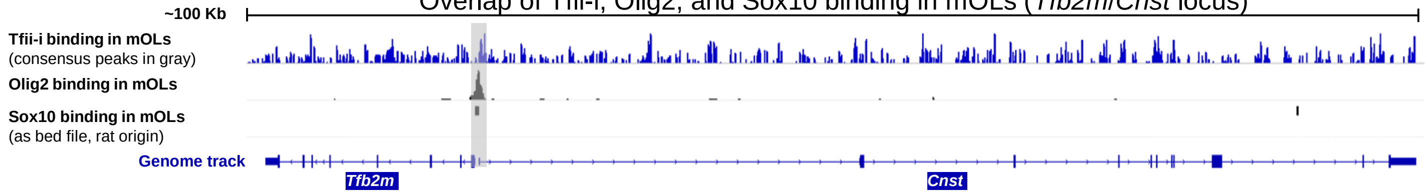

E

Overlap of Tfii-i, Olig2, and Sox10 binding in mOLs (*Rbm39* locus)

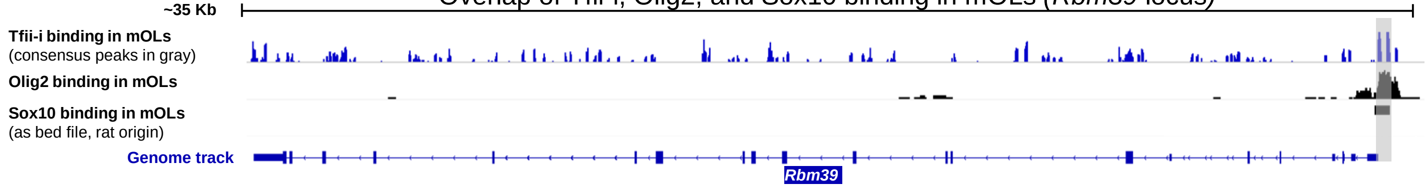

F

Motif analysis (20 bp window)

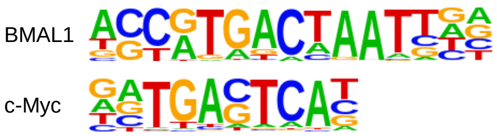

	-Log$_{10}$(p-value)
**BMAL1**	**4.014**
**c-Myc**	**3.518**

G

Tfii-i binding overlap with PTMH in mOLs

Histone	**Odds ratio**	***P*-value**
H3K36me3	2.054213	0.028819
H3K27ac	3.083854	0.143524
H3K4me3	1.510268	0.488894
H3K27me3	0	1

H

Evolutionary conservation ratios at Tfii-i peaks in mOLs

Peak #	Peak position in mouse genome (mm10) and length	Conserved fragment length	Conservation to human (%)
75 (Mbp)	Chr18:82555802-82556185<br>383 bp	104 bp	70.2
44 (Sox10 putative cRE)	Chr15:79110627-79111064<br>437 bp	174 bp	68.4

I

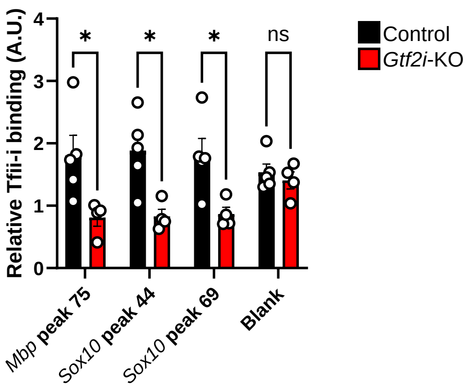

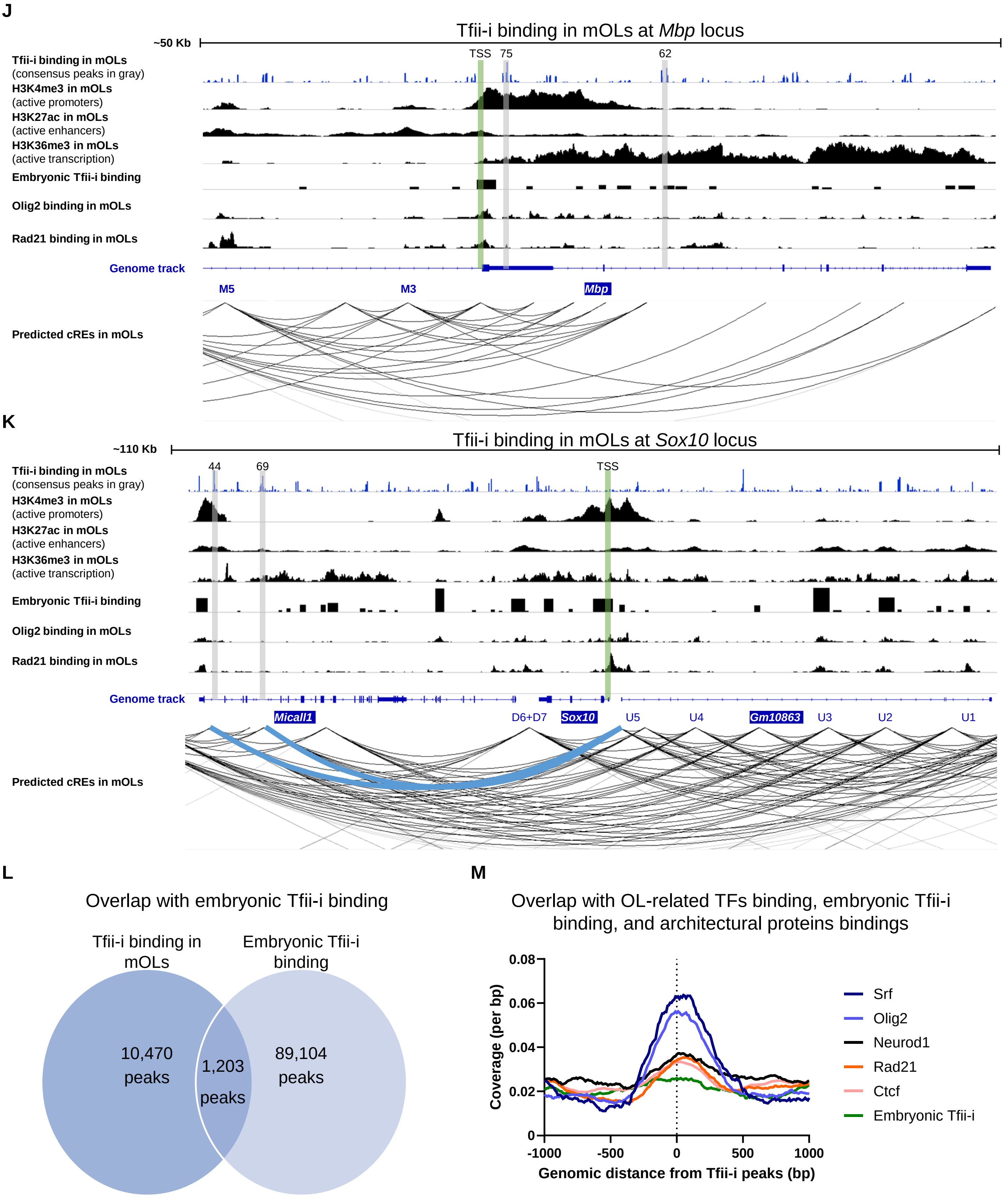
J
Tfii-i binding in mOLs at Mbp locus
~50 Kb
TSS
75
62
Tfii-i binding in mOLs
(consensus peaks in gray)
H3K4me3 in mOLs
(active promoters)
H3K27ac in mOLs
(active enhancers)
H3K36me3 in mOLs
(active transcription)
Embryonic Tfii-i binding
Olig2 binding in mOLs
Rad21 binding in mOLs
Genome track
M5
M3
Mbp
Predicted cREs in mOLs
K
Tfii-i binding in mOLs at Sox10 locus
~110 Kb
44
69
TSS
Tfii-i binding in mOLs
(consensus peaks in gray)
H3K4me3 in mOLs
(active promoters)
H3K27ac in mOLs
(active enhancers)
H3K36me3 in mOLs
(active transcription)
Embryonic Tfii-i binding
Olig2 binding in mOLs
Rad21 binding in mOLs
Genome track
Micall1
D6+D7
Sox10
U5
U4
Gm10863
U3
U2
U1
Predicted cREs in mOLs
L
Overlap with embryonic Tfii-i binding
Tfii-i binding in mOLs
Embryonic Tfii-i binding
10,470 peaks
1,203 peaks
89,104 peaks
M
Overlap with OL-related TFs binding, embryonic Tfii-i binding, and architectural proteins bindings
Coverage (per bp)
0.08
0.06
0.04
0.02
0
-1000
-500
0
500
1000
Genomic distance from Tfii-i peaks (bp)
Srf
Olig2
Neurod1
Rad21
Ctcf
Embryonic Tfii-i

Supplementary Figure 12. Supplementary ChIP-seq results and validation. **(A)** Table summarizing the number of peaks called in each of the five individual biological replicates. **(B)** Table summarizing the number of overlapping peaks across different combinations of replicates. **(C)** IGV track showing the inter-replicate variability of peak calling across all five biological replicates at the *Sox10* genomic locus. The upper track shows Tfii-i binding in mOLs (bed format), with consensus peaks 44 and 69 highlighted in gray. Other tracks peak calling per each replicate in the presented genomic locus. **(D-E)** Tfii-i, Olig2, and Sox10 binding overlap in mOLs. The upper track shows Tfii-i binding in mOLs with consensus peaks highlighted in gray. The second track shows Olig2 binding in mOLs[4], while the third track shows Sox10 binding in mOLs (bed format, from rat origin). **(D)** *Tfb2m* and *Cnst* genomic locus, **(E)** *Rbm39* locus. **(F)** Motif analysis of Tfii-i peaks within 20 bp window centered on peak summits reveals enrichment of E-box-related motifs, including BMAL1 and c-Myc. Tfii-i has previously been shown to bind to E-box motifs[1,2]. **(G)** Tfii-i colocalization analysis with mOLs-specific PTMH reveals significant enrichment of Tfii-i binding within H3K36me3-marked regions in mOLs (two-sided Fisher's exact test). **(H)** Sequences at peaks 75 and 44 are relatively evolutionarily conserved between mouse and human genomes. A 104 bp fragment within peak 75 (*Mbp* locus) shares 70.2% identity with the hg19 genome, while a 174 bp fragment within peak 44 (*Sox10* putative cRE) shares 68.4% identity. Analyses and visualizations were performed using the ECR browser[3]. **(I)** ChIP-qPCR validation of ChIP-seq results (n=5 control, n=4 *Gtf2i*-KO). Primers targeting consensus peaks 75 (*Mbp* locus), 44, and 69 (*Sox10* locus) confirmed reduced Tfii-i binding in *Gtf2i*-KO group compared to control group at peak 75 (two-sided t-test, P=0.035), peak 44 (two-sided t-test, P=0.013), and peak 69 (two-sided t-test, P=0.023). No significant change was observed at a negative control region (two-sided t-test, P=0.511). Data are presented as mean values ± SEM. **(J)** Tfii-i binding at *Mbp* genomic locus. The upper track shows Tfii-i binding in mOLs, with consensus peaks 75 and 62 highlighted in gray. The second, third, and fourth tracks show mOL-specific ChIP-seq data for H3K4me3, H3K27ac, and H3K36me3, respectively. The fifth and sixth tracks show Olig2 and Rad21 binding in mOLs[4], and the seventh track shows embryonic Tfii-i binding data[5]. The genome annotation track indicates known *Mbp* enhancers M3 and M5. The bottom track presents mOL-specific predicted cREs and their putative gene interactions, visualized as arcs. **(K)** Tfii-i binding at *Sox10* genomic locus. The upper track shows Tfii-i binding in mOLs, with consensus peaks 44 and 69 highlighted in gray. The second, third, and fourth tracks show mOL-specific ChIP-seq data for H3K4me3, H3K27ac, and H3K36me3, respectively. The fifth and sixth tracks show Olig2 and Rad21 binding in mOLs, and the seventh track shows embryonic Tfii-i binding data[5]. The genome annotation track indicates known *Sox10* enhancers. The bottom track presents predicted mOL-specific cREs, with Tfii-i peaks 44 and 69 located within intronic regions of the *Micall1* gene. These peaks align with cREs predicted to interact with the *Sox10* promoter, visualized as arcs and highlighted in blue. **(L)** Venn diagram showing the overlap between consensus Tfii-i binding sites identified in mOLs and previously published embryonic Tfii-i ChIP-seq data[5]. **(M)** Colocalization analysis of Tfii-i peaks in mOLs reveals substantial overlap with Srf and Olig2 binding, and limited overlap with embryonic Tfii-i, CTCF and Rad21 binding. *ns* - non-significant, * $P < 0.05$. Source data are provided as a Source Data file.

References (Supplementary Information)

1. Makeyev, A.V., et al., *Diversity and Complexity in Chromatin Recognition by TFII-I Transcription Factors in Pluripotent Embryonic Stem Cells and Embryonic Tissues.* PLOS ONE, 2012. **7**(9): p. e44443.
2. Roy, A.L., et al., *Cooperative interaction of an initiator-binding transcription initiation factor and the helix–loop–helix activator USF.* Nature, 1991. **354**(6350): p. 245-248.
3. Ovcharenko, I., et al., *ECR Browser: a tool for visualizing and accessing data from comparisons of multiple vertebrate genomes.* Nucleic Acids Research, 2004. **32**(suppl_2): p. W280-W286.
4. Bartosovic, M., M. Kabbe, and G. Castelo-Branco, *Single-cell CUT&Tag profiles histone modifications and transcription factors in complex tissues.* Nature Biotechnology, 2021. **39**(7): p. 825-835.
5. Kopp, N.D., et al., *Functions of Gtf2i and Gtf2ird1 in the developing brain: transcription, DNA binding and long-term behavioral consequences.* Human Molecular Genetics, 2020. **29**(9): p. 1498-1519.
