# Supplementary material for: Gtf2i-encoded transcription factor Tfii-i regulates myelination via Sox10 and Mbp regulatory elements
Source: Nat Commun. 2025 Sep 26;16:8518. doi: 10.1038/s41467-025-63500-4 (PMC12474925; doi:10.1038/s41467-025-63500-4)
Supplement: Supplementary file 2 — Description of Additional Supplementary Files [file 41467_2025_63500_MOESM2_ESM.pdf]

Description of Additional Supplementary Files

Supplementary Data 1: Proteomics data from CNS myelin fraction of Gtf2i-KO and control mice.

Supplementary Data 2: High confidence Tfii-i peaks in mOLs (ChIP-seq peaks).

Supplementary Data 3: 3C-qPCR and ChIP-qPCR primers.
