# Supplementary material for: Gtf2i-encoded transcription factor Tfii-i regulates myelination via Sox10 and Mbp regulatory elements
Source: Nat Commun. 2025 Sep 26;16:8518. doi: 10.1038/s41467-025-63500-4 (PMC12474925; doi:10.1038/s41467-025-63500-4)
Supplement: Supplementary file 5 — Supplementary Data 3 [file 41467_2025_63500_MOESM5_ESM.docx]

| **3C-qPCR primers** | | |
| --- | --- | --- |
| **Target** | **Sequence** | **Notes** |
| Constant primer | AACCTTTGCCCTGTACACAT | Located at *Sox10* promoter |
| 5kb | ACTCCCAGAAGATGCCAGAC |  |
| 35kb | TGTTACACTAGCGCTCCCTT |  |
| 45kb (peak 69) | CTTCCTCCTTTCCCTGCAGA | Used for whole cortex 3C-qPCR |
| 45kb (peak 69) V2 | GCTGATGGTTGTGTTGGACA | Used for OLs 3c-qPCR |
| 55kb (peak 44) | GCAGAAATTAGGAGCCAGGC | Used for whole cortex 3C-qPCR |
| 55kb (peak 44) V2 | TGAACCCCAGGCTCTAAGTG | Used for OLs 3c-qPCR |
| 65kb | GCCTTGGTGTAAATGGAGCA |  |
| 85kb | TCTACTTCCCCGCTGATGAC |  |
| **ChIP-qPCR primers** | | |
| **Target** | **F’** | **R’** |
| Peak 44 | F’-GCTATGGGACCCTGTGCTAA | R’-GTCAAGTCCAGCATCCTCCT |
| Peak 69 | F’-TGTATTTCAGCCAGGCATGC | R’-CGTCCTTGCCTTCACTCTGT |
| Peak 75 | F’-TGTGGCCATGGTTCAAGTTG | R’-ATGGAGGGAAAGGCACATCT |
