# Supplementary material for: Gtf2i-encoded transcription factor Tfii-i regulates myelination via Sox10 and Mbp regulatory elements
Source: Nat Commun. 2025 Sep 26;16:8518. doi: 10.1038/s41467-025-63500-4 (PMC12474925; doi:10.1038/s41467-025-63500-4)
Supplement: Supplementary file 6 — Reporting Summary [file 41467_2025_63500_MOESM6_ESM.pdf]

nature portfolio

Corresponding author(s): Prof. Boaz Barak

Last updated by author(s): Jul 22, 2025

Reporting Summary

Nature Portfolio wishes to improve the reproducibility of the work that we publish. This form provides structure for consistency and transparency in reporting. For further information on Nature Portfolio policies, see our Editorial Policies and the Editorial Policy Checklist.

Statistics

For all statistical analyses, confirm that the following items are present in the figure legend, table legend, main text, or Methods section.

n/a	Confirmed	
☐	☒	The exact sample size (*n*) for each experimental group/condition, given as a discrete number and unit of measurement
☐	☒	A statement on whether measurements were taken from distinct samples or whether the same sample was measured repeatedly
☐	☒	The statistical test(s) used AND whether they are one- or two-sided<br>*Only common tests should be described solely by name; describe more complex techniques in the Methods section.*
☐	☒	A description of all covariates tested
☐	☒	A description of any assumptions or corrections, such as tests of normality and adjustment for multiple comparisons
☐	☒	A full description of the statistical parameters including central tendency (e.g. means) or other basic estimates (e.g. regression coefficient) AND variation (e.g. standard deviation) or associated estimates of uncertainty (e.g. confidence intervals)
☐	☒	For null hypothesis testing, the test statistic (e.g. *F*, *t*, *r*) with confidence intervals, effect sizes, degrees of freedom and *P* value noted<br>*Give P values as exact values whenever suitable.*
☒	☐	For Bayesian analysis, information on the choice of priors and Markov chain Monte Carlo settings
☒	☐	For hierarchical and complex designs, identification of the appropriate level for tests and full reporting of outcomes
☒	☐	Estimates of effect sizes (e.g. Cohen's *d*, Pearson's *r*), indicating how they were calculated

Our web collection on statistics for biologists contains articles on many of the points above.

Software and code

Policy information about availability of computer code

Data collection	Noldus Ethovison XT software 14.0.1326, Noldus Observer XT
Data analysis	Noldus Ethovison XT software 14.0.1326, Prism GraphPad 10.5.0, ImageJ 1.54f

For manuscripts utilizing custom algorithms or software that are central to the research but not yet described in published literature, software must be made available to editors and reviewers. We strongly encourage code deposition in a community repository (e.g. GitHub). See the Nature Portfolio guidelines for submitting code & software for further information.

Data

Policy information about availability of data

All manuscripts must include a data availability statement. This statement should provide the following information, where applicable:

- Accession codes, unique identifiers, or web links for publicly available datasets
- A description of any restrictions on data availability
- For clinical datasets or third party data, please ensure that the statement adheres to our policy

The mass spectrometry proteomics data have been deposited to the ProteomeXchange Consortium via the PRIDE[203] partner repository with the dataset identifier PXD054341 [https://www.ebi.ac.uk/pride/archive/projects/PXD054341]. The ChIP-seq data have been deposited in the GEO accession database, under the accession identifier GSE285541 [https://www.ncbi.nlm.nih.gov/geo/query/acc.cgi?acc=GSE285541]. Source data are provided with this paper.

Research involving human participants, their data, or biological material

Policy information about studies with human participants or human data. See also policy information about sex, gender (identity/presentation), and sexual orientation and race, ethnicity and racism.

Reporting on sex and gender	N/A
Reporting on race, ethnicity, or other socially relevant groupings	N/A
Population characteristics	N/A
Recruitment	N/A
Ethics oversight	N/A

Note that full information on the approval of the study protocol must also be provided in the manuscript.

Field-specific reporting

Please select the one below that is the best fit for your research. If you are not sure, read the appropriate sections before making your selection.

[x] Life sciences [ ] Behavioural & social sciences [ ] Ecological, evolutionary & environmental sciences

For a reference copy of the document with all sections, see nature.com/documents/nr-reporting-summary-flat.pdf

Life sciences study design

All studies must disclose on these points even when the disclosure is negative.

Sample size	Sample size was chosen based on a previous similar study (Barak et al., Nature Neuroscience, 2019), and other studies in the field. Sample size used is based on common and accepted size in the field.
Data exclusions	Outliers of biological samples were calculated and excluded using the Grubbs' test (ESD method). Data exclusion on the 'Rotarod' test is described in the methods section.
Replication	Results were replicated by using more than one batch of test animals or were validated by other technical means and methods. All replications were successful.
Randomization	Experiments were randomized: mice order, items positions and samples loading were all randomized and counterbalanced.
Blinding	Experimenters were blinded during data collection and analysis, throughout all experiments.

Reporting for specific materials, systems and methods

We require information from authors about some types of materials, experimental systems and methods used in many studies. Here, indicate whether each material, system or method listed is relevant to your study. If you are not sure if a list item applies to your research, read the appropriate section before selecting a response.

Materials & experimental systems

n/a	Involved in the study	
[ ]	[x]	Antibodies
[x]	[ ]	Eukaryotic cell lines
[x]	[ ]	Palaeontology and archaeology
[ ]	[x]	Animals and other organisms
[x]	[ ]	Clinical data
[x]	[ ]	Dual use research of concern
[x]	[ ]	Plants

Methods

n/a	Involved in the study	
[ ]	[x]	ChIP-seq
[x]	[ ]	Flow cytometry
[ ]	[x]	MRI-based neuroimaging

Antibodies

Antibodies used	Commercial primary antibodies used in this study: rabbit anti-TFII-I (1:1000, catalog no. CST-4562S, Cell signaling), mouse anti-APC (Ab-7, CC1) (1:500, catalog no. OP80, Calbiochem), rabbit anti-Olig2 (1:1000, catalog no. AB9610, Sigma-Aldrich), rat anti-Pdgfrα (CD140a) (1:700, catalog no. 14-1401-82, Invitrogen), mouse anti-NeuN (1:1000, catalog no. MAB-377, Sigma-Aldrich), mouse anti-Sox10 (1:500, catalog no. SC-365692, Santa Cruz), rat anti-Mbp (1:500, catalog no. MAB386, Sigma-Aldrich), chicken anti-mCherry

(1:1000, catalog no. ab205402, Abcam), rabbit anti-NaV 1.6 (1:100, catalog no. ASC-009, Alomone labs), mouse anti-Caspr (1:50, catalog no. ab252535, Abcam), rabbit anti-Mog (1:500, catalog no. AB32760, Abcam), rabbit anti-tubulin (1:1000, catalog no. AB108342, Abcam), and rabbit anti-tubulin β4 (1:1000, catalog no. AB179509, Abcam). Secondary antibodies used in this study: goat anti-rabbit (1:1000, catalog no. ab150077, Abcam), goat anti-mouse (1:1000, catalog no. A11001, Invitrogen), goat anti-rat (1:1000, catalog no. ab150165, Abcam) conjugated to Alexa Flour 488. Goat anti-rabbit (1:1000, catalog no. A32732, Invitrogen), goat anti-chicken (1:1000, catalog no. A32932, Invitrogen), goat anti-mouse (1:1000, catalog no. A21424, Invitrogen) conjugated to Alexa Flour 555. Goat anti-rabbit (1:1000, catalog no. A21245, Invitrogen), goat anti-rat (1:1000, catalog no. A21247, Invitrogen) conjugated to Alexa Flour 647. Secondary antibodies for WB experiments: goat anti-rabbit (1:10000, catalog no. AP132P, Merck), and goat anti-rat (1:10000, catalog no. AP136P, Merck) antibodies.

Validation	All antibodies were validated by their manufacture for the applications they were used for. Primary antibodies were validated by 'No-First' staining.

Animals and other research organisms

Policy information about studies involving animals; ARRIVE guidelines recommended for reporting animal research, and Sex and Gender in Research

Laboratory animals	Mus musculus, C57Bl/6j one-month old, male mice were used. Gtf2i loxP mice (stock no. 000664; Jackson Laboratory) and Cnp-Cre mice (Lappe-Siefke C, et al., Nat Genet., 2003) were used.
Wild animals	The study did not involve wild animals.
Reporting on sex	Experiments were conducted specifically on males, similar to previous study (Barak et al., Nature Neuroscience, 2019). All primary cell cultures experiments were performed on both males and females.
Field-collected samples	The study did not involve samples collected from the field.
Ethics oversight	Experiments were approved by the institutional animal care and use committee of Tel Aviv University (approval number: 10-20-010, TAU-LS-IL-2408-137-2) and the Israel Ministry of Health. All efforts were made to minimize animal suffering and the number of animals used.

Note that full information on the approval of the study protocol must also be provided in the manuscript.

Plants

Seed stocks	N/A
Novel plant genotypes	N/A
Authentication	N/A

ChIP-seq

Data deposition

- [x] Confirm that both raw and final processed data have been deposited in a public database such as GEO.
- [x] Confirm that you have deposited or provided access to graph files (e.g. BED files) for the called peaks.

Data access links<br>*May remain private before publication.*	https://www.ncbi.nlm.nih.gov/geo/query/acc.cgi?acc=GSE285541
Files in database submission	54 raw data files (fastq.gz) and 10 processed data files (bed).
Genome browser session<br>(e.g. UCSC)	no longer applicable.

Methodology

Replicates	5 control samples, 4 Gtf2i-KO samples.
Sequencing depth	20M reads per sample, length of reads is 56 base pairs, paired-end.
Antibodies	Rabbit anti-TFII-I antibody (1:50, catalog no. CST-4562S, Cell signaling).
Peak calling parameters	Alignment of the raw fastq reads to the GRCm39 genome was performed using Bowtie2. Differential peak calling was performed using Macs2 (version 2.2.9.1) with the command line 'callpeak -t IP.bam -c Input.bam -g mm -f BAMPE -q 0.1 --nolambda --keep-dup all –B', where immunoprecipitate (IP) over input scores were calculated. Additional manual filtration was performed to exclude peaks with; q-value > 0.01, fragment size larger than 600bp and peaks that were mapped to mitochondrial DNA.
Data quality	The Bedtools package (using the intersect and cluster tools) was employed to identify Tfii-i consensus peaks, defined as those present

in at least three out of five samples in the control group. Peaks were classified as "lost" if no overlap was observed between the KO group and the Tfii-i consensus peaks. Respective heatmaps were generated by custom R scripts and the Complexheatmap R package. HOMER package was used for peaks annotation and motif enrichment analysis. Motif analysis was performed using HOMER with a 20bp window. Genomic tracks were generated in IGV.

Software

Alignment of the raw fastq reads to the GRCm39 genome was performed using Bowtie2. Differential peak calling was performed using Macs2 (version 2.2.9.1) with the command line 'callpeak -t IP.bam -c Input.bam -g mm -f BAMPE -q 0.1 --nolambda --keep-dup all –B', where immunoprecipitate (IP) over input scores were calculated. Additional manual filtration was performed to exclude peaks with; q-value > 0.01, fragment size larger than 600bp and peaks that were mapped to mitochondrial DNA. The Bedtools package (using the intersect and cluster tools) was employed to identify Tfii-i consensus peaks, defined as those present in at least three out of five samples in the control group. Peaks were classified as "lost" if no overlap was observed between the KO group and the Tfii-i consensus peaks. Respective heatmaps were generated by custom R scripts and the Complexheatmap R package. HOMER package was used for peaks annotation and motif enrichment analysis. Motif analysis was performed using HOMER with a 20bp window. Genomic tracks were generated in IGV. UCSC liftover tool was used to lift genome annotations from the mm39 genome to the mm10 genome.

Magnetic resonance imaging

Experimental design

Design type: N/A

Design specifications: N/A

Behavioral performance measures: N/A

Acquisition

Imaging type(s): Diffusion tensor imaging

Field strength: 7 T

Sequence & imaging parameters: The MRI protocol included diffusion imaging acquisition with a diffusion-weighted spin-echo echo-planar imaging pulse sequence. Acquired volumes were 18 slices, each 0.6 mm thick, with the following parameters: resolution of 0.175 mm × 0.175 mm2 (matrix size, 128 × 96), repetition time of 3000 ms, echo time of 25 ms, Δ/δ was 10/2.5 ms, 4 echo-planar imaging segments, and 15 non-collinear gradient directions with a single b-value shell at 1000 s/mm2 and 3 images with a b-value of 0 s/mm2 (b0 image). The DTI acquisition took 10 min and 48 seconds.

Area of acquisition: Whole brain

Diffusion MRI: [x] Used [ ] Not used

Parameters: The MRI protocol included diffusion imaging acquisition with a diffusion-weighted spin-echo echo-planar imaging pulse sequence. Acquired volumes were 18 slices, each 0.6 mm thick, with the following parameters: resolution of 0.175 mm × 0.175 mm2 (matrix size, 128 × 96), repetition time of 3000 ms, echo time of 25 ms, Δ/δ was 10/2.5 ms, 4 echo-planar imaging segments, and 15 non-collinear gradient directions with a single b-value shell at 1000 s/mm2 and 3 images with a b-value of 0 s/mm2 (b0 image). The DTI acquisition took 10 min and 48 seconds.

Preprocessing

Preprocessing software

All diffusion MRI analysis was performed in ExploreDTI, and included the following steps:
1. Motion and distortion correction to correct for possible motion- and susceptibility-induced artifacts.
2. Transformation into atlas space via non-linear registration and extraction of atlas space FA and MD per mouse brain.
3. Whole-brain fiber tracking with 0.175 mm × 0.175 mm × 0.175 mm seed voxel resolution; minimal FA and stropping criteria for tracking: FA > 0.1; maximal 30° tracking angle allowed. Tracking step size: 0.175 mm.
4. The reconstructed number of fibers was taken for statistical analysis between groups.

Normalization: We used a population-specific template.

Normalization template: We used a population-specific template.

Noise and artifact removal: Motion and distortion correction to correct for possible motion- and susceptibility-induced artifacts.

Volume censoring: N/A

Statistical modeling & inference

Model type and settings: Voxel-wise independent two-sided t-tests

Effect(s) tested: Genotypic effect was studied, where 2 groups were compared.

Specify type of analysis: [ ] Whole brain [ ] ROI-based [x] Both

Anatomical location(s) | regions of interest were defined having into account the regions showing significant differences in the whole brain analyses.

Statistic type for inference (See Eklund et al. 2016) | The reconstructed number of fibers was taken for statistical analysis between groups.

Correction | FWE and FDR corrections were used

Models & analysis

n/a	Involved in the study
☒	☐ Functional and/or effective connectivity
☒	☐ Graph analysis
☒	☐ Multivariate modeling or predictive analysis
